# Early Steps of a Thymic Tumor in SV40 Transgenic Mice: Hyperplasia of Medullary Epithelial Cells and Increased Mature Thymocyte Numbers Disturb Thymic Export

**DOI:** 10.1080/10446670310001593532

**Published:** 2002-12

**Authors:** Bernadette Nabarra, Catherine Martinon, Cécile Godard, Florence Vasseur, Geoffroy de Ribains, Lucile Miquerol, Axel Kahn, Sophie Ezine

**Affiliations:** 1 INSERM U.345 Institut Necker Paris Cedex 15 75730 France; 2 INSERM U.238 CEA. Grenoble Cedex 9 38054 France; 3 INSERM U.129 CHU Cochin-Port-Royal Paris 75014 France

## Abstract

Bone marrow progenitors migrate to the thymus, where they proliferate and differentiate into immunologically competent T cells. In this report we show that mice transgenic for SV40 T and t antigens under the control of the L-pyruvate kinase promoter develop, in a first step, thymic hyperplasia of both thymocytes and epithelial cells. Morphological studies (histology, immunohistolabeling and electron microscopy) revealed modifications of the thymic microenvironment and gradual expansion of medullary epithelial cells in 1 month-old mice, taking over the cortical region. Then, a thymic carcinoma develops. Two-color labeling of frozen sections identified the transgene in medullary epithelial cells. Flow cytometry analysis demonstrated a marked increase in mature CD4^+^ and CD8^+^ thymocytes in adult mice (39±10×10^6^ in transgenic mice and 12±5×10^6^ in age-matched controls). Furthermore, thymocyte export was disturbed.


*Developmental Immunology*, December 2002 Vol. 9 (4), pp. 223–231

Taylor & Francis healthsciences

### Early Steps of a Thymic Tumor in SV40 Transgenic Mice: Hyperplasia of Medullary Epithelial Cells and Increased Mature Thymocyte Numbers Disturb Thymic Export*

BERNADETTE NABARRA[a], CATHERINE MARTINON[b], CÉCILE GODARD[a], FLORENCE VASSEUR[a], GEOFFROY DE RIBAINS[c], LUCILE MIQUEROL[c], AXEL KAHN[c] and SOPHIE EZINE[a,†]

[a]*INSERM U.345, Institut Necker, 75730, Paris Cedex 15, France;* [b]*INSERM U.238, CEA, 38054, Grenoble Cedex 9, France;* [c]*INSERM U.129, CHU Cochin-Port-Royal, 75014, Paris, France*

Bone marrow progenitors migrate to the thymus, where they proliferate and differentiate into immunologically competent T cells. In this report we show that mice transgenic for SV40 T and t antigens under the control of the L-pyruvate kinase promoter develop, in a first step, thymic hyperplasia of both thymocytes and epithelial cells. Morphological studies (histology, immunohistolabeling and electron microscopy) revealed modifications of the thymic microenvironment and gradual expansion of medullary epithelial cells in 1 month-old mice, taking over the cortical region. Then, a thymic carcinoma develops. Two-color labeling of frozen sections identified the transgene in medullary epithelial cells. Flow cytometry analysis demonstrated a marked increase in mature $CD4^+$ and $CD8^+$ thymocytes in adult mice ($39 \pm 10 \times 10^6$ in transgenic mice and $12 \pm 5 \times 10^6$ in age-matched controls). Furthermore, thymocyte export was disturbed.

*Keywords*: Immunohistolabeling; Ultrastructure; Thymic medullary tumor; L-pyruvate kinase promoter

*Abbreviations*: DN, double negative $CD4^-CD8^-$; DP, double positive $CD4^+CD8^+$; SP, single positive $CD4^+CD8^+$; SV40, simian virus 40; Tag, T antigen; TCR, T cell receptor; Tg, transgenic

### INTRODUCTION

In the thymic microenvironment, the epithelial compartment is organized into cortical and medullary zones that mediate different aspects of thymocyte differentiation. The different processes controlling the growth and organization of the epithelial compartment depend largely on cell interactions involving thymocytes and stromal cells for selection, differentiation and maturation of T cells (Duijvestin *et al.*, 1981; Weissman *et al.*, 1982; Kendall, 1986; Nabarra, 1987; 1991a; Marrack, 1988; von Bohmer, 1988; Sprent *et al.*, 1988; Brekelmans and van Ewijk, 1990; van Ewijk, 1991; Boyd *et al.*, 1993). Furthermore, thymic stroma cells included also non-epithelial cells (bone marrow-derived cells) as macrophages and interdigitated cells (IDC) which are also involved in these process.

In this milieu, thymocytes are in symbiotic developmental relationship involving the different stromal cells and various signaling molecules, cytokines, cytokine receptors and chemokines (Zlotnik and Moore, 1995; Norment and Bevan, 2000). T cell precursors migrating from the bone-marrow to the thymus undergo an ordered differentiation process. After different migrations steps across the organ, mature T cells are generated and exit the thymus for the periphery (Scollay *et al.*, 1980; Pénit, 1986). Migration and homing are partly dependent on adhesion molecules (Imhof *et al.*, 1991; Aurrand-Lions *et al.*, 1996). Virtually nothing is known about the precise thymic location from which mature thymocytes emigrate from the thymus to peripheral lymphoid organs. However, the cortico-medullary junction has been suggested important in this process in association with maintenance of a normal architecture.

The studies of all these parameters, particularly elucidation of the cross-talk between thymocytes and epithelial cells (van Ewijk *et al.*, 1994; Pénit *et al.*, 1996), is difficult to appreciate in steady state conditions. Nevertheless, the studies of modification and disruption of the thymic microenvironment organization in different pathologic mice, and recently of genetically engineered mice, appeared to be a good approach in relation with

*Presented at the Proceedings of the 4th Germinal Center Conference, June 2002, Groningen, The Netherlands.

†Corresponding author. Tel.: +33-1-40-61-53-65. Fax: +33-1-40-61-55-80. E-mail: ezine@necker.fr

ISSN 1044-6672 print © 2002 Taylor & Francis Ltd
DOI: 10.1080/10446670310001593532

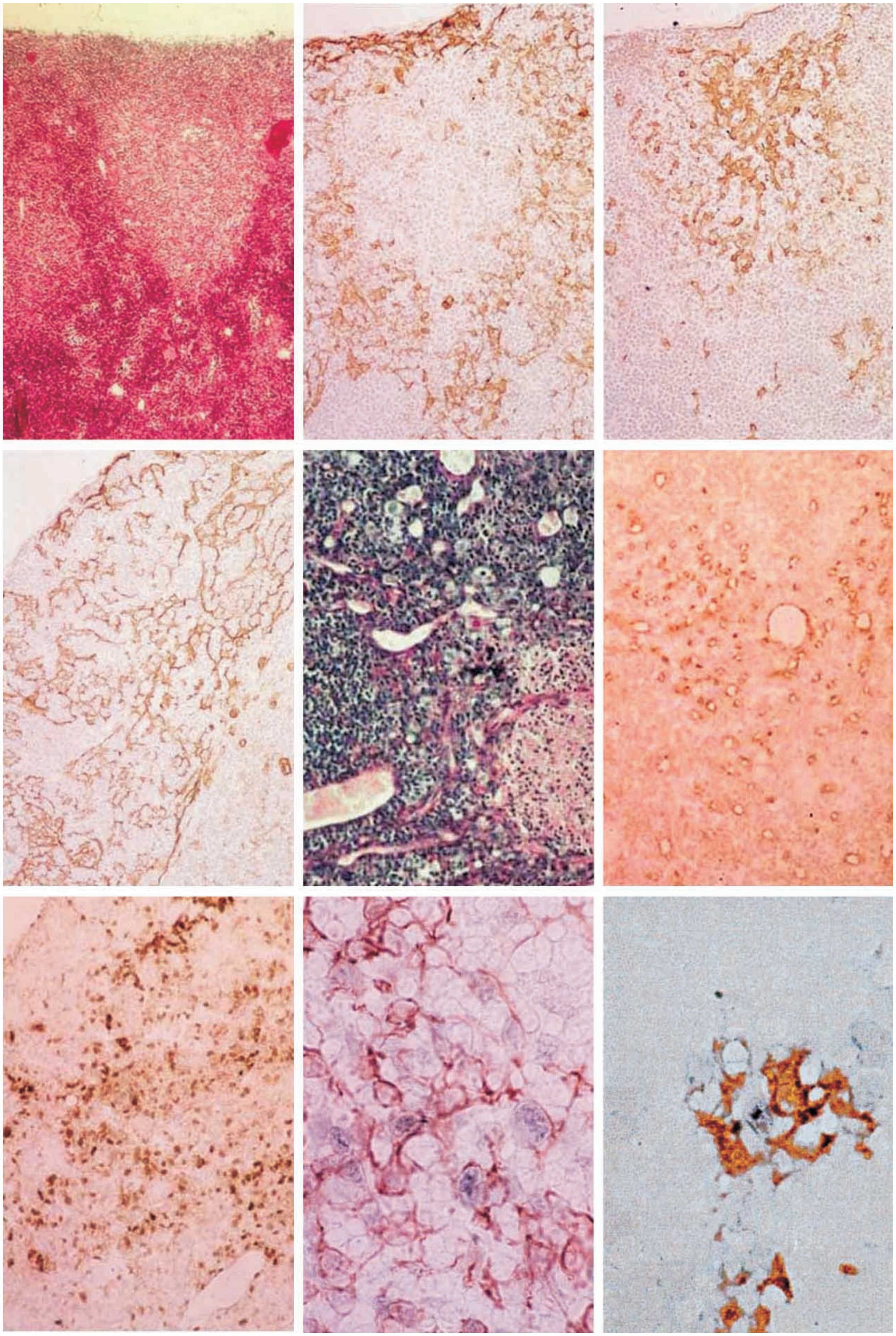

observations of cellular modifications, alterations in the developmental program and phenotype of thymocytes (Rouse and Weissman, 1981; Kendall, 1986; Nabarra and Dardenne, 1991b; Naquet *et al.*, 1999).

In this way, both naturally occurring and experimentally-induced tumors were used as models for dissecting *in vivo* these different sequences of T cell education, and disruption of thymic stroma.

Transgenic model of pure thymic tumor is rarely described. Today only one team has described a thymic carcinoma issued of an extanded thymic hyperplasia in Tg mice made with SV40 Simian Virus T antigen 40 (SV40) associated with it own promoter (Park *et al.*, 1996; Lee *et al.*, 1998).

We have generated a second mouse model with SV40 T and t Ag with a different promoter (L-pyruvate kinase). SV12 transgenic mice develop early in life a massive thymic hyperplasia concerning both thymocytes and epithelial cells. Immuno-histological studies, confirmed by electron microscopy studies, demonstrate a large hyperplasia of expanded medullary epithelial cells, carrying the transgene. With time, large angiogenesis, numerous cellular atypies concerning the cytoplasm and the nucleus and a large thymic epithelial tumor of carcinoma type with formation of necrotic nodules are observed (Nabarra, in preparation). Thymocyte differentiation was stable, but resulted in increased numbers of mature single-positive (SP) thymocytes. The impact of this modified microenvironment on thymic maturation and export is discussed.

### RESULTS

### Generation of SV12 Transgenic Mice

The structure of the fusion genes was described previously and has been published (Miquerol *et al.*, 1996). Transgenic mice were generated by microinjecting DNA into the pronucleus of fertilized mouse eggs. Newly integrated sequences were identified after hybridization of tail DNA with a probe for SV40 T Ag. Three transgenic mouse lines, with different transgene copy numbers (SV12, SV19, SV25), which all carried SV40 T antigen genes in the germ line, were obtained. The SV12 line is characterized by a rapidly growing thymic tumor: thymus weight is 1.6 g at 16 weeks, compared to 0.05 g in age-matched controls. The SV10 line develops a thymic tumor of the same size as that in SV12 mice, but much later (around 8 months); the SV25 line is intermediate, as the thymic tumor develops at 6 months. It is unclear why such promoter can lead to thymic tumors. We have focused this study on the SV12 line.

### SV12 Thymic Microenvironment (1–4 Months)

The morphological aspect of the thymic stromal cells, in the first months of life, is described here, at the stages of large hyperplasia and beginning of tumor development when the malignancy is not obvious.

### Medullary Epithelial Cell Expansion and Disorganization of the Thymic Stroma

We observed from the first weeks of life, a large hyperplasia concerning both the stroma cells and the lymphocytes without alterations of the architecture. At 2 months, the thymic microenvironment was disorganized regarding location of cortical and medullary areas. Histological staining showed a marked development of the medulla, largely extended to the capsule, alternating with narrow and remained area of the cortical zone, giving an inverted picture (Fig. 1a) compared to the normal thymus. Numerous lymphoid cells were present in all these stromal areas. High mitotic activity was detected.

Labeling with MTS10 antibody shows several large positive medullary zones going under the capsula and shifting in unusual location reduced cortical spots labeled with MTS5 antibody (Fig. 1b–d). Later on, and on classical histologic staining, the presence of round and clear cells with a large nucleus, scattered and/or in small groups was observed around 4 months, associated with fewer thymocytes but with always numerous mitoses. The vascular network was modified and many neo-vessels appeared in the hyperplasic parenchyma as focused toward the formations of clear cells (Fig. 1e). These numerous neo-vessels are labeled with CD31 (specific of endothelial vascular cells) (Fig. 1f) showing an important angiogenesis process.

Ultrastructural examination confirm the abnormal composition of the thymic microenvironment with an increasing number of epithelial cells of medullary type (type III and IV immature cells) (Nabarra, 1987;1991a) (Fig. 2a–c). In a second step some unclassified cells

FIGURE 1 (a) Histology of SV12 mice at 2 months showing the altered distribution of the medullary zone (clear staining) and the cortical zone, with more numerous strongly colored lymphocytes. Trichrome staining. X400. (b) MTS-5 labeling showing the modified and reduced repartition of the "cortical" areas in 2 month-old SV12 mice. X400. (c) MTS-10 labeling of medullary epithelial cells of the modified thymic microenvironment in 2 month-old SV12 mice. X400. (d) Labeling with MTS-10 antibody at 4 months, showing groups of medullary epithelial cells extending under the capsule and, in down right area, a large "white" zone (MTS10 labeling) with a few isolated MTS-$10^+$ cells. X400. (e) Histological aspect at 4 months in an area of thymic hyperplasia, with numerous clear round cells crossed by numerous neovessels organized toward "nodular" cellular clear areas (down right). Trichrome staining. X400. (f) Aspect at 4 months in SV12 mice, showing a large increase in the number of small vessels labeled with CD31 X400. (g) Low magnification of the thymic microenvironment in 2 month-old SV12 mice showing, scattered in the stroma, numerous cells labeled with monoclonal antibodies against SV40 T antigen. X400. (h) Double immunolabeling with SV40 and MTS-10 antibodies, revealing majority of cells with jointly the transgene stained in blue on nucleus and medullary epithelial cells stained brown by peroxidase. X1000. (i) Double immunolabeling with SV40 T and N418 antibodies. The blue nucleus with a strongly labeled nucleolus is not associated with peroxidase brown cells corresponding to the interdigitated cells (N418). X1000.

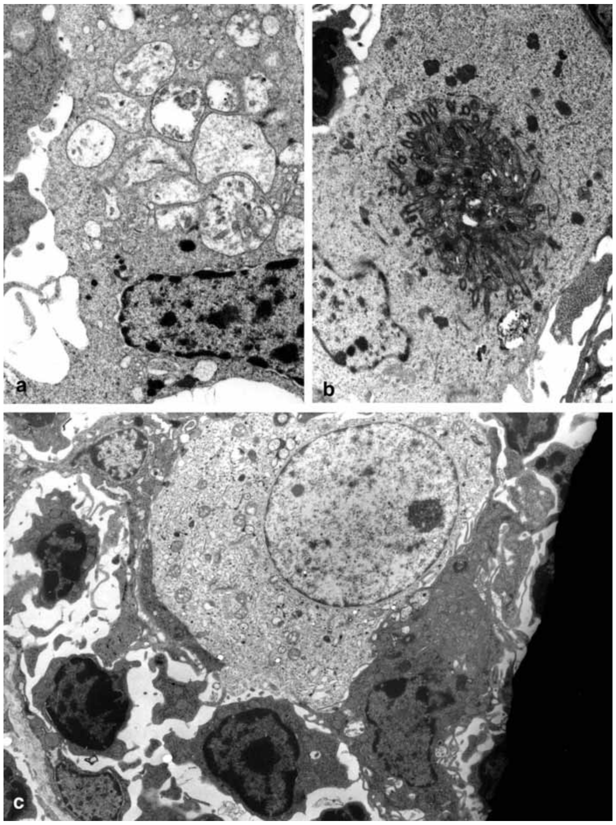

FIGURE 2 Ultrastructural aspect of the different types of thymic medullary epithelial cells: (a) Type II epithelial cell (arrow head: tonofilament) with the characteristic intracytoplasmic “alveolar labyrinth”. Impregnated by uranyl acetate and lead citrate. X12500. (b) Type III epithelial cell with an intracytoplasmic cavity having a lumen full of dense contains and bordered by microvillosities and numerous cilia. Impregnated by uranyl acetate and lead citrate. X12000. (c) Immature epithelial cells (Type IV) with very light tonofilaments and small desmosomes in a clear cytoplasm (arrow head). Immature nucleus with a clear coarse chromatin and dense reticuled nucleolus are present. Impregnated by uranyl acetate and lead citrate. X16500.

having the epithelial characteristics (tonofilaments and desmosomes) but without other specific morphological characteristics appeared to be undifferentiated (Fig. 3a). Numerous lymphocytes and lymphoblasts with a larger cytoplasm containing a high number of ribosomes were present especially at 2 months. Mitosis were frequent in both epithelial cells and lymphocytes (Fig. 3a). Numerous vessels in formation with turgescent endothelium were found in this modified thymic parenchyma (Fig. 3b).

Finally from 4 months, numerous cellular atypies concerning both cytoplasm and nucleus were noted (not shown).

### Transgene Expression is Limited to Medullary Epithelial Cells

To determine which subset of epithelial cells expressed the transgene, immunohistolabeling studies were performed with anti-T SV40 antibody on frozen sections. Two- and four-month-old SV12 mice showed dense staining of numerous cells, mostly grouped in large zone but a few number was scattered throughout the thymus parenchyma (Fig. 1g). High magnification revealed labeling of dense clumps in the nucleus of large cells, and sometimes a discreet staining of the stellate cytoplasm. This clearly indicated that epithelial cells were $SV40^{+}$.

Dual immunolabeling with anti-SV40 T and MTS-10 antibodies revealed that the transgene (staining blue clumps in the nucleus) was present in most medullary epithelial cells (labeled by brown peroxidase) (Fig. 1h). Thus, $SV40^{+}MTS\text{-}10^{+}$ medullary epithelial cells were observed in large areas of the modified thymic architecture. Very few $SV40^{+}$ cells were not labeled by the MTS10 antibody.

Dual labeling with SV40 and N418 (chiefly a marker of interdigitated cells) showed that interdigitated cells were increased in number and scattered throughout the modified microenvironment, and that none of these cells expressed SV40 (Fig. 1i).

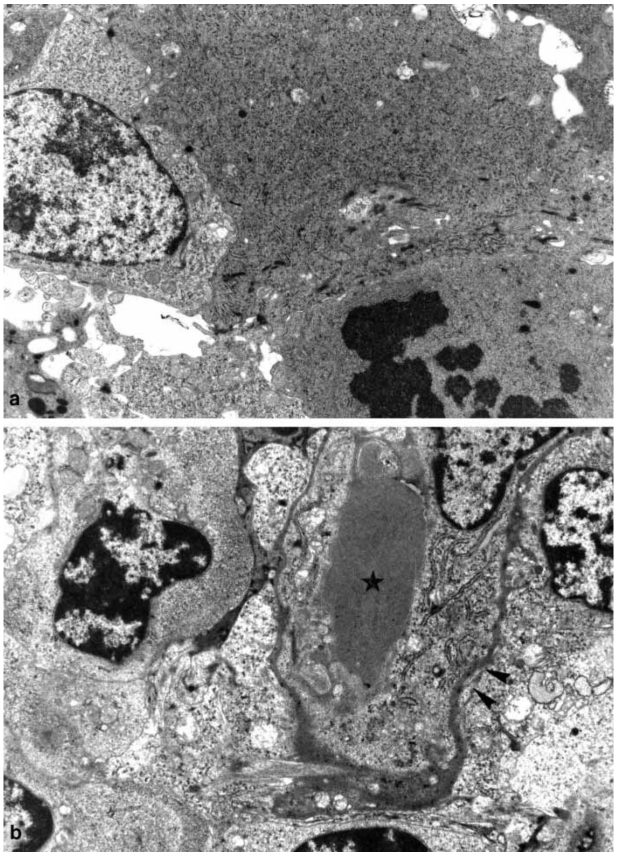

FIGURE 3 (a) Large and extended epithelial cytoplasm (presence of tonofilaments) with numerous ribosomes and without morphological characteristics. They appear less differentiated. At the bottom of the micrograph, an epithelial cell (forming desmosomes) in mitosis. Impregnated by uranyl acetate and lead citrate. X18000. (b) Head of a new vessel with red blood cells (*), turgescent endothelium and basal membrane in formation (arrow head). Epithelial cells and lymphoblasts are present in the environment. Impregnated by uranyl acetate and lead citrate. X22000.

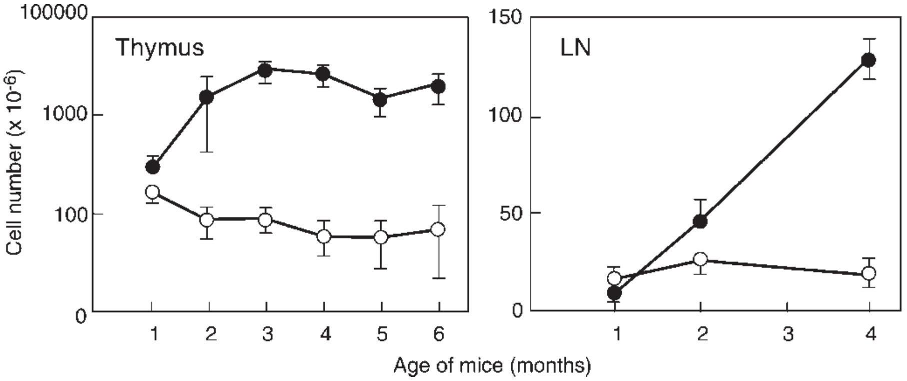

FIGURE 4 Cellularity in SV12 thymus and lymph nodes with age. Age-dependent cellularity in thymus (a) and lymph nodes (b) in SV12 mice and littermate controls. Mean values ± SD of 6 to 8 animals. SV12 (●), CBA littermates (○).

### Lymphoid Hyperplasia is Present Early in Age with Increased Mature Thymocyte Numbers

Thirty-two transgenic mice and 31 controls were studied between 4 weeks and 6 months of age. The phenotype of the mice had been stable for more than 18 generations. Most mice die around 5–6 months of age most probably by asphyxia due to thymic outgrowth. Fig. 4 shows the kinetics of thymic cellularity in SV12 and control mice: between 6 and 8 weeks SV12 mice show a significant increase in total cell numbers; maximum thymus size is reached around 12 weeks and remains constant thereafter. No difference in proliferation (assessed by BrdUrd incorporation) was observed between SV12 and control thymuses (data not shown). As shown in Fig. 4, lymphadenopathy was observed after 2 months of age in SV12 mice, and T and B cell numbers were increased in all lymph nodes. Thymocyte subsets were then analyzed to further characterize the phenotype of the SV12 transgenic line.

Thymocyte differentiation was very stable in SV12 mice throughout the study. All four thymic subsets were always present. The relative proportion of the most immature population ($CD4^-CD8^-$) was similar in SV12 and controls, but the $CD4^+CD8^+$ (DP) population was decreased in SV12 (Fig. 5a, (a and e)). Marked CD25 expression by the DP subset was always found (Fig. 5a, (c and g)); thus, it reveals incomplete down regulation of CD25 from the triple negative 3 (TN3 subset, $CD25^+$ $CD44^-$) subset for an unknown reason. Analysis of mature thymic subsets revealed larger numbers of $TCR\alpha\beta^+$ thymocytes in SV12 (Fig. 5a, (f)) than in aged-matched controls (Fig. 5a, (b)).

The numbers of $CD4^+$- and $CD8^+$-$TCR\alpha\beta^+$ mature T cells became significantly higher in SV12 than in controls around 2 months of age (Fig. 4 and 5b). Down-regulation of HSA antigen expression reflected the complete maturation of $TCR\alpha\beta^+$ thymocytes (Fig. 5a, (d and h)). These populations showed normal expression of activation markers (CD69, CD44, CD25, CD62L) (not shown). Moreover, Vβ subsets among mature T cells were normal (not shown). The accumulation of mature thymocytes could reflect defective homing and/or specific outgrowth of this subset, but thymocyte proliferative activity was normal in SV12 mice (not shown).

### Thymic Export is Disturbed in SV12 Mice

The marked thymic hyperplasia observed as early as 6 weeks and involving both thymocytes and epithelial cells, together with the increase in mature thymocyte numbers prompted us to analyze the output of mature T cells. Injection of fluorescein into the thymic lobes allowed us to analyze thymic export to lymph nodes. As shown in Table I, at 1 month and 5 months of age the number of thymic emigrants was higher in SV12 mice than in controls. However, the ratio of thymic emigrants to mature thymocytes revealed a very low level of thymocyte release in SV12 mice compared to controls, and this defect increased with age. Thus, thymic export is altered in SV12 mice since more thymocytes were expected to emigrate.

### DISCUSSION

In SV12 Tg mice, the thymus becomes hyperplasic concerning both stromal cells and lymphocytes. We show a very large extension of the medullary epithelial cells which carried exclusively the transgene. In this modified microenvironment mature single positive $CD4^+$ and $CD8^+$ thymocyte numbers increase. Furthermore, analysis of thymic export in SV12 mice shows a significant reduction in T cell emigration. Altogether, these results suggest that the altered medullary environment in SV12 transgenic mice supports normal thymocyte differentiation but disturbs thymic export.

We will focus our discussion on the thymocyte populations and their relationship with the different cellular components of the stromal microenvironment, especially the medullary epithelial cells in the hyperplasic stage.

We note firstly that in several SV40 T Tg mouse models, thymic hyperplasia is sometimes associated with

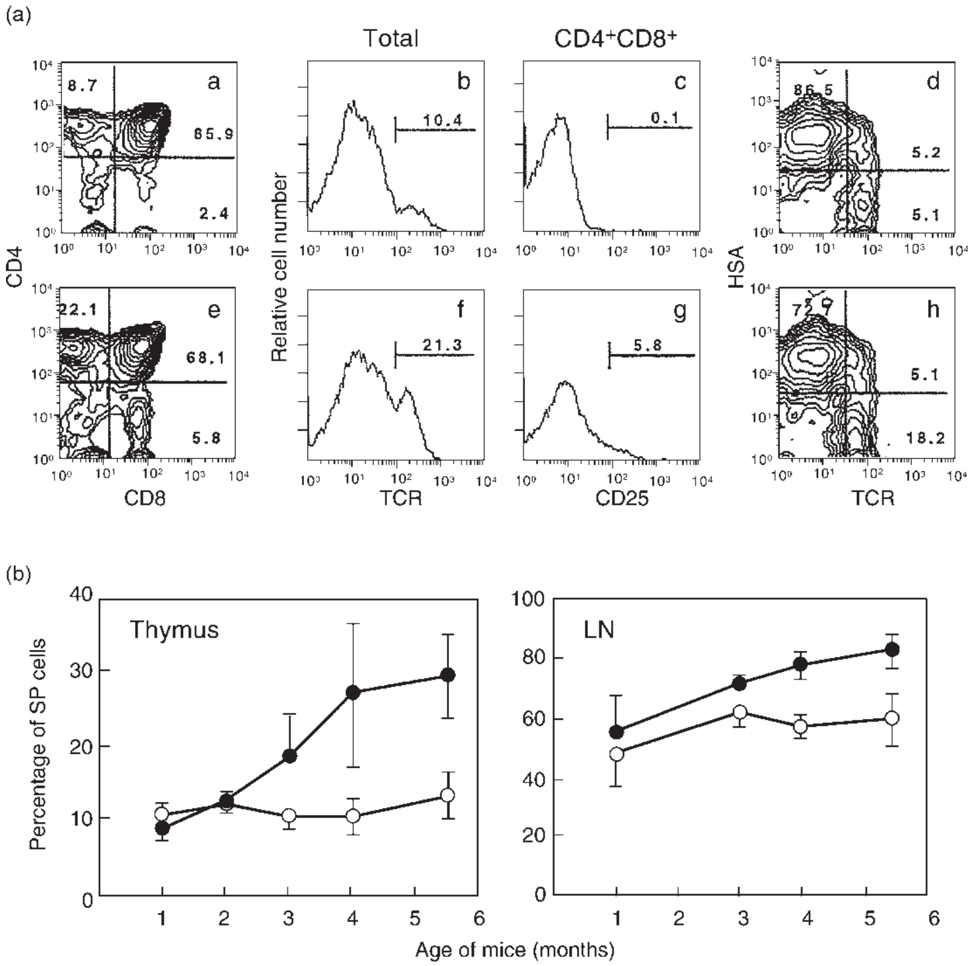

FIGURE 5 Thymic differentiation in SV12 Mice. (a) Comparison of thymocyte subpopulations of CBA (a–d) and SV12 (e–h) mice. Thymocytes were analyzed by two-color cytometry for expression of TcR vs. HSA (d, h) or by three-color cytometry for CD4, CD8 and TcR (a, b, e, f) or CD25 (c, g). In b and f, the profiles show TcR expression in total thymocyte population; in c and g, the profiles show CD25 expression on gated $CD4^+CD8^+$ cells. Numbers indicate percentages of cells. (b) Age-dependent percentage of single positive cells ($CD4^+CD8^-$ and $CD8^+CD4^-$) in thymus (a) and lymph nodes (B) in SV12 mice and littermate controls. $CD4^+CD8^-$ and $CD8^+CD4^-$ phenotypes are visualized by double staining of thymocytes with PE anti-CD4 and FITC anti-CD8 antibodies. Mean values ± SD of 3–6 animals. SV12 (●), CBA littermates (○).

tumor development in various other organs (Brinster *et al.*, 1984; Palmiter *et al.*, 1985; Botteri *et al.*, 1987; Reynolds *et al.*, 1988; Messing *et al.*, 1988; Teitz *et al.*, 1995). In these reports, the hyperplasia appeared lymphocytic with an eventual alteration of the stroma rarely studied. It is noted an increase in lymphocyte numbers with normal subset representation. These authors suppose that the lymphocyte proliferation is in relation with the stromal cell modifications. In the model of thymic tumor reported by Park *et al.* (1996) an increase in total lymphocyte

TABLE I Thymic export is altered in SV12 transgenic mice

| Age of mice | | | Number of thymic emigrants per day ( $\times 10^{-6}$) | Ratio of thymic emigrants/mature thymocytes |
|---|---|---|---|---|
| 1 month | Experiment 1 | $Tg^-$ | 0.26 | 1/68 |
| | | $Tg^+$ | 0.79 | 1/228 |
| | Expériment 2 | $Tg^-$ | 0.82 | 1/19 |
| | | $Tg^-$ | 0.63 | 1/26 |
| | | $Tg^+$ | 1.34 | 1/15 |
| | | $Tg^+$ | 1.15 | 1/26 |
| 5 months | Experiment 1 | $Tg^-$ | 0.73 | 1/9 |
| | | $Tg^-$ | 2.44 | 1/6 |
| | | $Tg^+$ | 4.04 | 1/107 |
| | Experiment 2 | $Tg^-$ | 0.45 | 1/5 |
| | | $Tg^+$ | 7.52 | 1/79 |
| | | $Tg^+$ | 1.92 | 1/183 |

At the age indicated, mice received an intrathymic injection of FITC as described in Materials and Methods section. Eighteen hours later, thymus, spleen and lymph nodes were analyzed by FACS for the presence of $FITC^+$ cells.

number, chiefly in mature T cells, is also associated to stromal cell modifications but not characterized.

In our model, expression of the transgene by medullary epithelial cells is associated with marked proliferation of this cell type. This large proliferation is responsible for the disruption of the thymic architecture, with loss of organization, disruption of the cortico-medullary junction and marked expansion of medullary cells. Blockade of different tumor suppressor factors might induce epithelial proliferaton (Hanaban *et al.*, 1985; Ludlow, 1993; Robles *et al.*, 1994). The epithelial cells appeared directly involved in the observed lymphocytic hyperplasia. Indeed, bone marrow transplantation from transgenic mice to irradiated non transgenic recipients leads to the development of a normal thymus (data not shown). Cytokines and growth factors are produced by transformed epithelial cells (Moll *et al.*, 1992; Fass *et al.*, 1993), and the thymocyte hyperplasia observed in the SV12 line could be due to these stimulating factors, which could act by enhancing cell growth and/or survival.

The exact role of thymic stroma cells in T cell maturation and selection of the T cell repertoire is unclear. Thymocyte development in SV12 mice is remarkably stable, with an age-related increase in mature thymocyte numbers; this subset is increased in percentage and absolute number. The microenvironment contributes to the maintenance of mature thymocytes in specific cross-talk interactions. Thus, T cells or/and their immediate stromal partners might be defective. Abnormal thymocyte antigen expression may prevent thymic export. However, CD62L expression is normal on SV12 thymocytes (data not shown); therefore, involvement of other molecules in thymic emigration and/or of endothelium-thymocyte interactions might play a role (Yagi *et al.*, 1996). The absence of any detectable defect at the lymphocyte level strongly points to stromal malfunction. However, it does not interfere with thymocyte differentiation; mechanical constraints due to a disorganized network of epithelial cells are probably involved. Reports of SV40-induced cortical epithelial tumor development also showed an increase in mature T cells (Park *et al.*, 1996; Lee and Seo, 1996). It is assumed that all maturing thymocytes exit from the cortico-medullary junction, thus this process might be hindered by disruption of this junction during medullary (our report) or cortical (Lee and Seo, 1996) epithelial expansion. The exit failure described in the SV12 model is present as early as 1 month, before peripheral T cell hyperplasia.

However, we cannot exclude that other mechanisms (as homeostatic mechanisms) play a role. For example, in another study (Volkmann *et al.*, 1996), thymic hyperplasia with increased production of thymocytes and increased export of T cells results in a normal size of the peripheral T cell pool. Thus, disregulation of the export is largely dependent on the *in situ* thymic environment. Another hypothesis considers that thymocytes could also act by enhancing cell growth and/or survival on the epithelium, which may respond to signals produced by the overproliferating lymphocytes in these tumors. This can be supported by works claiming that the development of the medullary zone involves the presence of mature T cells (Surh *et al.*, 1992). But it is accepted that hyperplasia is not an autonomous property of mutant T cells and it is rather likely that the modified microenvironment involves inappropriate epithelial factors or cell–cell contacts and the increase, as a consequence, of the number of lymphocytes in the thymic stromal compartment.

Our study shows a new model among SV40 transgenic mice. For the first time we clearly show that the transgene is expressed in the thymic medullary epithelium, and that mature thymocyte accumulation is due to a disturbed thymic export. Thus, the expanded medullary epithelium might have lost a critical organization directing the export process. SV12 mice should prove useful for studies of the mechanism underlying thymocyte emigration.

### MATERIALS AND METHODS

### Mice

The SV12 transgenic line was generated at Cochin Hospital by A. Kahn's team (Miquerol *et al.*, 1996). The transgene encodes T and t antigens from SV40 (2.7 Kb fragment of the simian virus genome) under the control of the pyruvate kinase promoter (Cla I/EcoRv fragment) and the SV40 enhancer (270–95 nt fragment). Three independent transgenic lines were obtained (SV25, SV19 and SV12) according the copy number integrated in the genome. SV12 females are infertile, and the line is thus maintained by crossing heterozygous SV12 Tg males with CBA females. Controls are non transgenic littermates. Mice were tested for the presence of the transgene by means of PCR on DNA extracted from tail biopsy specimens. The primer SVS3 and PK-L sequences are:

- SVS3: 5′GCATCCCAGAAGCCTCCAAAG3′,

- PK-L:5′GCA ACGTAGCAGCATGGAAG3′.

Tail DNA was incubated in 50 μl of Taq buffer solution (ATGC) with dNTP (1 mM), 0.5 unit of Taq polymerase and 0.1 μg of each primer. The amplification sequences consisted of 8 min at 94°C then 30 cycles at 92°C (30″), 55°C (30″) and 72°C (1′). The last cycle is followed by incubation at 72°C for 5′.

### Monoclonal Antibodies

The following antibodies were used for cytometric analysis: anti-CD4 (clone GK 1.5), anti-CD8 (clone 53-6.7), anti-αβ-TCR (clone H57-597), anti-CD25 (clone PC61), anti-CD44 (clone Pgp1), anti-CD24 (clone J11d) and anti-B220 (clone RA3-6B2). They were purchased from Pharmingen (Becton Dickinson, Grenoble, France)

or prepared in our laboratory from hybridomas and used as directly coupled to fluoresceine thyocyanate (FITC) or phycoerythrin (PE) or cychrome (Cy) and if coupled to biotin, detected by streptavidin-Cy.

The following antibodies were used for immunolabeling studies with different dilutions: MTS5 (1:20) (against cortical epithelial cells) (Boyd *et al.*, 1993), MTS10 (1:100) (against medullary epithelial cells) (Boyd *et al.*, 1993), anti-CD31 (1:200) (specific for endothelial vascular cells), anti-SV40 T coupled to biotin (1:800) (Pharmingen); N418 (pure) (specific for interdigitated cells) was a kind gift from G. Kraal (Vrije University, Amsterdam, The Netherlands).

The second step uses mouse anti-rat biotin (MAR) (1:40, Immunotech), mouse anti-hamster biotin (MAH) (1:100, Pharmingen) or TSA-biotin system (NEN Life Science Products).

The third step is 3-3′ diaminobenzidine (Fast DAB, Sigma) for MTS5, MTS10 and CD31 and Elite amplification kit (Vector Laboratories) for N418. Streptavidin-peroxidase (Vector Laboratories) was also used to detect biotinylated antibodies.

### Morphological Techniques

Dissected thymic tissues were immediately fragmented and prepared for three techniques.

*Histology*: The thymuses of 12 mice in each age group (1–6 months) were fixed in Bouin's solution for 12 h and included in paraffin, then sections of 3 microns were stained with hematoxylin eosin, Masson trichrome and PAS.

*Immunohistology*: Fragments were placed in cryovials (Greiner) and quickly placed in liquid nitrogen. Frozen sections (5 microns) were fixed for 10 min in acetone at room temperature and air-dried. For single-color labeling, in the first step, antibodies were incubated for 1 h at room temperature. For the second step, the incubation is of 30 min, followed by the third step described previously.

Double labeling is performed with SV40-biotin (1:40) in the first step or MTS10 (1:100) or N418 (pure) in a second step followed by Elite amplification kit (for SV40), MAR-bio (for MTS10) or MAH (for N418); last step includes VSG (Vector Laboratories) for SV40 or Elite amplification kit and Nova Red (Vector Laboratories) for MTS10 and N418. For all, rehydratation and washes were performed with PBS + 5% FCS. Slides were mounted in Entellan.

*Electron microscopy*: Small fragments of different thymuses were fixed in 1.6% glutaraldehyde in Sorensen's buffer + 9% NaCl, pH 7.4, for 1 h. After three washes in the same buffer they were post-fixed in 1% osmium tetroxide for 1 h. After washing, the fragments were dehydrated in a series of alcohol and propylene oxide and embedded in EPON 812 resin (Polysciences). Ultrathin (700 Å) sections were cut, places on copper grids and impregnated with uranyl acetate and lead citrate for examination in a Philips EM 300 electron microscope. Twelve mice at each age (1–6 months) were used for morphologic studies.

### Emigration Assay

The test was performed as described by Scollay *et al.* (1980), except that 20–50 μl of FITC solution at 1 mg/ml in PBS was intrathymically injected into one thymic lobe, owing to the large size of the thymuses. Mice were killed 16 h later and their thymus, spleen and lymph nodes were removed. Between 20 and 50% of thymocytes were $FITC^+$.

### *Acknowledgements*

The authors thank R. Boyd for gift of antibodies in the initial phases of this work. We gratefully thank D.D. Young for English corrections, B. Chemani for the photographic work and S. Hamon and M. Netter for the preparation of the manuscript.

### *References*

Aurrand-Lions, M., Galland, F., Bazin, H., Zakharvey, V.M., Imhof, B.A. and Naquet, P. (1996) "Vanin-1, a novel GPI-linked perivascular molecule involved in thymus homing", *Immunity* **5**, 391–405.

Botteri, F., van den Putten, H., Wong, D., Sauvage, C. and Evans, R. (1987) "Unexpected thymic hyperplasia in transgenic mice harboring a neuronal promoter fused with SV40 large T antigen", *Mol. Cell Biol.* **7**, 3178–3184.

Boyd, R.L., Tucek, C.L., Godfrey, D.I., Izon, D.J., Wilson, T.J., Davidson, N.J., Bean, A.G., Ladyman, H.M., Ritter, M.A. and Hugo, P. (1993) "The thymic microenvironment", *Immunol. Today* **14**, 445–459.

Brekelmans, P. and van Ewijk, W. (1990) "Phenotypic characterization of murine thymic microenvironments", *Semin. Immunol.* **2**, 13–24.

Brinster, R., Chen, H., Messing, A., Levine, A. and Palmiter, R. (1984) "Transgenic mice harboring SV40 T antigen genes develop characteristic brain tumors", *Cell* **37**, 367–379.

Duijvestijn, A. and Hoefsmit, E. (1981) "Ultrastructure of the rat thymus. The microenvironment of T lymphocyte maturation", *Cell Tissue Res.* **218**, 279–282.

Fass, J., Rothstein, J., Kreider, B., Rovera, G. and Knowles, B. (1993) "Phenotypically diverse mouse thymic stromal cell lines which induce proliferation and differentiation of hematopoietic cells", *Eur. J. Immunol.* **23**, 1201–1214.

Hanaban, D. (1985) "Heritable formatin of pancreatic beta-cell tumors in transgenic mice expressing recombinant insulin/SV40 oncogenes", *Nature* **315**, 115–122.

Imhof, B.A., Ruiz, P., Hesse, B., Palacios, R. and Dunon, D. (1991) "EA-1, a novel adhesion molecule involved in the homing of progenitor T lymphocytes to the thymus", *J. Cell Biol.* **114**, 1069–1071.

Kendall, M. (1986) "Functional anatomy of the thymic microenvironment", *J. Anat.* **147**, 95–106.

Lee, W.H. and Seo, J.S. (1996) "Alterations of the thymic selection process in transgenic mice expressing SV40 large T antigen", *Int. J. Cancer* **67**, 399–404.

Lee, S.S., Park, W.Y., Chi, J.G., Seo, J.W., Kim, J.I., Kim, C.W., Park, S.H., Khang, S.K., Cho, K.J., Seo, J.S. and Jang, J.J. (1998) "Thymic epithelial tumor progression in an SV40 T transgenic mouse model", *Virchows Arch.* **432**, 33–42.

Ludlow, J. (1993) "Interactions between SV40 large tumor antigen and the growth suppressor proteins pRB and p53", *FASEB. J.* **7**, 866–871.

Marrack, P., Lo, D., Brinster, R., Palmiter, R., Burkly, L., Flavell, R.H. and Kapler, J. (1988) "The effect of thymus microenvironment on T cell development and tolerance", *Cell* **53**, 627–634.

Messing, A., Pinkert, C., Palmiter, R. and Brinster, R. (1988) "Developmental study of SV40 large T antigen expression in transgenic mice and choroid plexus nepolasia", *Oncogene Res.* **3**, 87–91.

Miquerol, L., Cluzeaud, F., Porteu, A., Alexandre, Y., van de Walle, A. and Kahn, A. (1996) "Tissue specificity of L-pyruvate kinase transgenes results from the combinatorial effect of proximal promoter and distal activator regions", *Genet. Expr.* **5**, 315–320.

Moll, J., Eibel, H., Botteri, F., Sansig, G., Regnier, C. and van der Putten, H. (1992) "Transgenes encoding mutant simian virus 40 large T antigens unmask phenotypic and functional constraints inthymic epithelial cells", *Oncogene* **7**, 2175–2187.

Nabarra, B. and Andrianarison, I. (1987) "Ultrastructural studies of thymic reticulum. I. Epithelial components", *Thymus* **9**, 95–100.

Nabarra, B. and Andrianarison, I. (1991) "Ultrastructural studies of thymic reticulum. II. Non-epithelial components", *Thymus* **17**, 39–43.

Nabarra, B. and Dardenne, M. (1991) "Evidence of abnormal microenvironment in mice with autoimmune diseases", In: Imhof, Berrih and Ezine, eds, *Lymphatic Tissue and In Vivo Immune Response* (M. Dekker, New York), p 665.

Naquet, P., Naspetti, M. and Boyd, R. (1999) "Development, organization and function of the thymic medulla in normal, immunodeficient or autoimmune mice", *Semin. Immunol.* **11**, 47–55.

Norment, A. and Bevan, N. (2000) "Role of chemokines in thymocyte development", *Semin. Immunol.* **12**, 445–455.

Palmiter, R., Chen, H., Messing, A. and Brinster, R. (1985) "SV40 enhancer and large T antigen are instrumental in development of choroid plexus tumors in transgenic mice", *Nature* **316**, 457–460.

Park, W.Y., Kim, J.I., Shim, E.H., Lee, W.H., Kim, S.H., Seo, J.W., Jang, J.J. and Seo, J.S. (1996) "Development of thymic carcinoma in transgenic mice expressing SV40 T antigen", *Cancer Lett.* **107**, 293–300.

Pénit, C. (1986) "*In vivo* thymocytes maturation: BUdR labeling of cycling thymocytes and phenotypes analysis of their progeny support the single lineage model", *J. Immunol.* **137**, 2115–2121.

Pénit, C., Lucas, B., Vasseur, F., Rieker, T. and Boyd, R.L. (1996) "Thymic medulla epithelial cells acquire specific markers by post-mitotic maturation", *Dev. Immunol.* **55**, 25–36.

Reynolds, R., Hockzema, G., Vogel, J., Hinrichs, S. and Jay, G. (1988) "Multiple endocrine neoplasia induced by the promiscuous expression of a viral oncogene", *Proc. Natl Acad. Sci. USA* **85**, 3135–3139.

Robles, M., Symonds, H., Chen, J. and van Dyke, T. (1994) "Induction versus progression of brain tumor development: differential functions for pRB and p53 targeting domains of simian virus 40 T antigen", *Mol. Cell Biol.* **14**, 2686–2698.

Rouse, R. and Weissman, I. (1981) "Microanatomy of the thymus: its relationship to cell differentiation", Microenvironment in Hematopoietic and Lymphoid Differentiation (Ciba Foundation, Pitman Medical), pp 161–177.

Scollay, R., Butcher, E. and Weissman, I. (1980) "Thymus cells migration. Quantitative aspects of cellular traffic from the thymus to the periphery in mice", *Eur. J. Immunol.* **10**, 210–218.

Sprent, J., Lo, D., Gao, E.K. and Ron, Y. (1988) "T cell selection in the thymus", *Immunol. Rev.* **101**, 173–178.

Surh, C., Ernst, B. and Sprent, J. (1992) "Growth of epithelial cells in the thymic medulla is under the control of mature T cells", *J. Exp. Med.* **176**, 611–616.

Teitz, T., Chang, J., Wai Kan, Y. and Yen, T. (1995) "Thymic epithelial neoplasm in transgenic mice expressing SV40 T antigen under the control of an eruthroid-specific enhancer", *J. Pathol.* **177**, 309–315.

van Ewijk, W. (1991) "T cell differentiation is influenced by thymic microenvironment", *Annu. Rev. Immunol.* **9**, 591–615.

van Ewijk, W., Shores, E. and Singer, A. (1994) "Cross-talk in the mouse thymus", *Immunol. Today* **15**, 214–217.

Volkmann, A., Döffinger, R., Rüther, U. and Kyewski, B.A. (1996) "Insertional mutagenesis affecting programmed cell death leads to thymic hyperplasia and altered thymopoiesis", *J. Immunol.* **156**, 136–145.

von Bohmer, H. (1988) "The developmental biology of T lymphocytes", *Annu. Rev. Immunol.* **6**, 309–313.

Weissman, I., Rouse, R., Kyewski, B., *et al.* (1982) "Thymic lymphocyte maturation in the thymic microenvironment", *Behring Inst. Mitt.* **70**, 242–248.

Yagi, H., Matsumoto, M., Nakamura, M., Makino, S., Zuzuki, R., Harada, M. and Itoh, T. (1996) "Defect of thymocyte emigration in a T cell deficiency strain (CTS) of the mouse", *J. Immunol.* **157**, 3412–3419.

Zlotnik, A. and Moore, T.A. (1995) "Cytokine production and requirements during T-cell development", *Curr. Opin. Immunol.* **7**, 206–213.

